# Multicenter assessment and longitudinal study of the prevalence of antibodies and related adaptive immune responses to AAV in adult males with hemophilia

**DOI:** 10.1038/s41434-024-00441-5

**Published:** 2024-02-14

**Authors:** Ingrid Pabinger, Mila Ayash-Rashkovsky, Miguel Escobar, Barbara A. Konkle, María Eva Mingot-Castellano, Eric S. Mullins, Claude Negrier, Luying Pan, Kavitha Rajavel, Brian Yan, John Chapin

**Affiliations:** 1 Clinical Division of Hematology and Hemostaseology, Department of Medicine I, Medical University of Vienna, Vienna, Austria; 2 Takeda Development Center Americas Inc, Cambridge, MA USA; 3 University of Texas Health Science Center, McGovern Medical School and Gulf States Hemophilia and Thrombophilia Center, Houston, TX USA; 4 BloodWorks Northwest, Seattle, WA USA; 5 Division of Hematology, University of Washington School of Medicine, Seattle, WA USA; 6 Hospital Regional Universitario de Málaga, Málaga, Spain; 7 Hospital Universitario Virgen del Rocio, Sevilla, Spain; 8 Division of Hematology, Cincinnati Children’s Hospital Medical Center and University of Cincinnati-College of Medicine, Cincinnati, OH USA; 9 UR4609 Hemostase & Thrombose, University Lyon 1, Lyon, France

**Keywords:** Haematological diseases, Biological techniques

## Abstract

Adeno-associated virus (AAV) based gene therapy has demonstrated effective disease control in hemophilia. However, pre-existing immunity from wild-type AAV exposure impacts gene therapy eligibility. The aim of this multicenter epidemiologic study was to determine the prevalence and persistence of preexisting immunity against AAV2, AAV5, and AAV8, in adult participants with hemophilia A or B. Blood samples were collected at baseline and annually for ≤3 years at trial sites in Austria, France, Germany, Italy, Spain, and the United States. At baseline, AAV8, AAV2, and AAV5 neutralizing antibodies (NAbs) were present in 46.9%, 53.1%, and 53.4% of participants, respectively; these values remained stable at Years 1 and 2. Co-prevalence of NAbs to at least two serotypes and all three serotypes was present at baseline for ~40% and 38.2% of participants, respectively. For each serotype, ~10% of participants who tested negative for NAbs at baseline were seropositive at Year 1. At baseline, 38.3% of participants had detectable cell mediated immunity by ELISpot, although no correlations were observed with the humoral response. In conclusion, participants with hemophilia may have significant preexisting immunity to AAV capsids. Insights from this study may assist in understanding capsid-based immunity trends in participants considering AAV vector-based gene therapy.

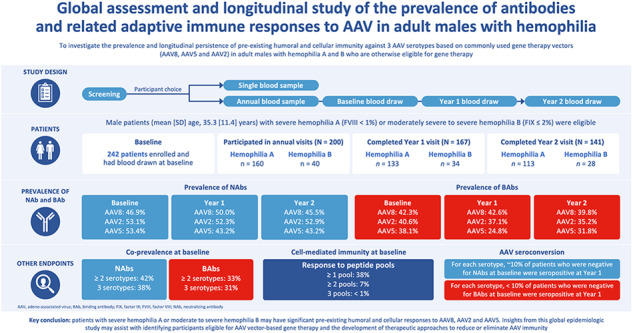

## Introduction

Hemophilia is an X-linked congenital bleeding disorder caused by a loss of function mutation, which results in the deficiency of coagulation factors VIII (FVIII; hemophilia A) or IX (FIX; hemophilia B) [1]. Gene transfer holds substantial promise as a curative intervention for congenital bleeding disorders, and adeno-associated virus (AAV) vector-based gene therapies have made significant progress in clinical trials [1–4].

Recombinant AAV vectors have unique gene-transfer properties that make them attractive vehicles for delivering functional genes, including the ability to target specific tissues, systemic intravenous delivery, and a favorable safety profile [5]. However, exposure to wild-type AAV serotypes can lead to humoral and cellular immune responses directed against the AAV capsid antigens. One example is the development of anti-AAV neutralizing antibodies (NAbs) from exposure to wild-type AAV, which inhibits recombinant AAV vectors from transducing target tissues and prevents sustained transgene expression [6–10]. Therefore, hemophilia clinical trial participants with preexisting humoral AAV immunity are ineligible for most AAV-based gene therapy clinical studies and approved products [2–4, 11].

Previous seroprevalence studies have been conducted to understand the prevalence of preexisting immunity against AAV [12], but not all studies have provided a longitudinal, multiyear description of persistent NAbs against multiple AAV serotypes. Furthermore, reports on the prevalence of preexisting cell-mediated immunity, as determined using an enzyme-linked immunospot (ELISpot) assay, are limited in this population. The aim of this study was to investigate the prevalence and longitudinal persistence of preexisting humoral and cellular immunity against three AAV serotypes based on commonly used gene therapy vectors (AAV8, AAV5, and AAV2) in adults with hemophilia A and B who are otherwise eligible for gene therapy.

## Methods

### Study design

This prospective, epidemiological, longitudinal study examined the seroprevalence of preexisting immunity to AAV in adult males with hemophilia receiving treatment at hemophilia treatment centers across the United States and the European Union (NCT03185897). This was a noninterventional study designed to collect blood samples either for one visit or annually for four visits (Supplementary Fig. S1), according to participant preference at enrollment. The first participant was enrolled in June 2017 and the last participant completed the study in March 2021.

This study was conducted in accordance with the International Council for Harmonization Guideline for Good Clinical Practice, the Declaration of Helsinki, and applicable international, national, and local regulatory requirements. Institutional review boards (IRBs), independent Ethics Committees for each site, and Central IRBs approved this protocol. All participants provided written informed consent.

### Participants

Eligible participants were male (age 18–75 years) and had established severe hemophilia A (plasma FVIII activity <1%) or moderately severe to severe hemophilia B (plasma FIX activity ≤2%). Participants were excluded if they had any bleeding disorders other than hemophilia A or B, or laboratory evidence of having developed inhibitors to FVIII or FIX protein at any time (≥0.6 Bethesda units on any single test). Participants were also excluded if they were receiving systemic immunosuppressive, cytotoxic, or monoclonal Ab therapy (including FVIII mimetics), or antiviral treatments for hepatitis C. Participants with HIV or prior viral hepatitis were permitted in this trial. Participants with a history of an immune deficiency other than HIV, and those with lymphocyte or plasma cell malignancies, were also excluded. Exposure to immunoglobulins (Igs) or plasma transfusion ≤120 days prior to blood draw at enrollment was not permitted.

### Objectives

The primary objective of this study was to assess the seroprevalence of NAbs to AAV (AAV8, AAV5, and AAV2) in adults with severe hemophilia A or moderately severe to severe hemophilia B.

Secondary objectives included determining the proportion of participants with AAV8 or AAV2 NAb titers ≥1:5; examining the presence of binding Abs (BAbs) to AAV8, AAV5, and AAV2 and the presence of cell-mediated immunity to AAV (AAV capsid-specific T-lymphocyte responses); and identifying potential correlations between circulating AAV8 NAbs, BAbs, and other humoral and cell-mediated immune responses to AAV. In addition, the co-prevalence of NAbs and BAbs to AAV2, AAV8, and AAV5 were assessed at all visits. Exploratory objectives prospectively measuring AAV seroconversion and Ab titer fluctuations.

### Study measures

All study assessments were performed by a central laboratory. Anti-AAV8, anti-AAV2, and anti-AAV5 NAbs in serum were measured at baseline and at each visit using a cell-based in vitro transduction inhibition assay, to assess the potential for serum samples collected from a study participant to inhibit luciferase marker gene transfer in cell culture by AAV, as previously described [11, 13]. Briefly, serial 2-fold dilutions of heat-inactivated participants’ serum or negative control serum were mixed 1:1 with rAAV8-, rAAV2-, or rAAV5-luciferase reporter vector and incubated for 1 h at 37 °C and 5% CO_2_. This preincubated mix was then added to Huh7 cells (JCRB Cell Bank, JCRB0403) which have been pre-incubated with Ad-DL309 helper virus and incubated overnight at 37 °C and 5% CO_2_. Luciferase activity was quantified using a luminometer. The highest dilution of the participant’s serum that resulted in inhibition of ≥50% of luciferase activity (compared with the negative pooled human serum control) was recorded as the NAb titer. The assay cut point was determined by statistical analysis of data generated from six runs of 50 lots of commercially sourced samples from healthy participants. Seropositivity was defined as a result equal to or greater than the assay cut point, with a minimum required dilution (MRD) of 1:5. The NAb titer was defined as the reciprocal of highest sample dilution with response above or equal to the assay cut point.

Anti-AAV8 BAbs (IgG and IgM), anti-AAV2 IgG BAbs, and anti-AAV5 IgG BAbs were measured using an enzyme-linked immunosorbent assay with horseradish peroxidase anti-human IgG Abs or IgM Abs as the detection Abs. Briefly, AAV8, AAV2, and AAV5 capsids were immobilized on a microwell plate. Participant samples, along with a positive control Ab, were exposed to the immobilized capsid to allow binding. After washing, horseradish peroxidase–conjugated anti-human IgG Abs (Biorad, STAR106P) or horseradish peroxidase–conjugated anti-human IgM Abs (Biorad STAR145P, for anti-AAV8 BAb only) were then added to the plate and incubated. Following plate washing, the chromogenic substrate 3,3'5,5’-tetramethylbenzidine was added. The reaction was terminated using sulfuric acid and outputs were measured using colorimetric assessment with a plate reader. The presence of BAbs was determined by comparing the control sample signal to a statistically derived assay cut point. Seropositivity was defined as a result equal to or greater than the assay cut point, with an MRD of 1:20. The BAb titer was defined as the reciprocal of highest sample dilution with response above or equal to the assay cut point. The MRD of 1:20 for BAb assays were selected based on the balance of assay sensitivity and matrix interference to meet the study needs. The 1:20 dilution reduced assay background signals while maintaining sufficient assay sensitivity. The screening assay cut point was established with a 5% false positive rate using cut point data generated from 50 lots of normal human plasma.

Cell-mediated immune responses against AAV8 peptide antigens were determined using a peripheral blood mononuclear cell-based ELISpot assay for interferon (IFN)-γ secretion. The presence of T cells that reacted to three different AAV8 antigen peptide pools or phytohemagglutinin was measured. Peripheral blood mononuclear cells were isolated using Ficoll-Hypaque gradient centrifugation and cultured with media (negative control), AAV8 peptides, or phytohemagglutinin (positive control) for 20–24 h. IFN-γ spots were quantified using the CTL immunospot analyzer with Immunospot software and processed with SpotMap software. Samples with a signal ≥3× background and >60 cells/million peripheral blood mononuclear cells were defined as positive. A single positive AAV8 antigen pool response was considered sufficient to report the ELISpot assay as positive.

### Statistical analysis

A sample size of up to 250 participants was planned, including a group of approximately 200 participants with hemophilia A and approximately 50 participants with hemophilia B. The sample size was not selected based on statistical justifications. Assuming the range of seroprevalence of NAb to AAV8 is 30–40% in the adult male population, the confidence interval width ranges from 0.131 to 0.140 for hemophilia A participants with a sample size of 200, and from 0.267 to 0.284 for hemophilia B participants with a sample size of 50.

All data processing, summarization, and analyses used the SAS^®^ software package, version 9.4. There was no statistical hypothesis test for the 2 different participant groups (hemophilia A and B). Categorical summaries are presented as number and percentage of participants, with 95% confidence intervals (CIs). The Clopper-Pearson method was used to calculate the exact binomial 95% CIs of proportions. Continuous summaries are displayed using summary statistics (numbers of subjects, mean, standard deviation, median, minimum, maximum, 1st quartile, 3rd quartile). No missing data analysis techniques were performed, and data were analyzed and presented as recorded in the database.

## Results

### Participant disposition

**Fig. 1:**
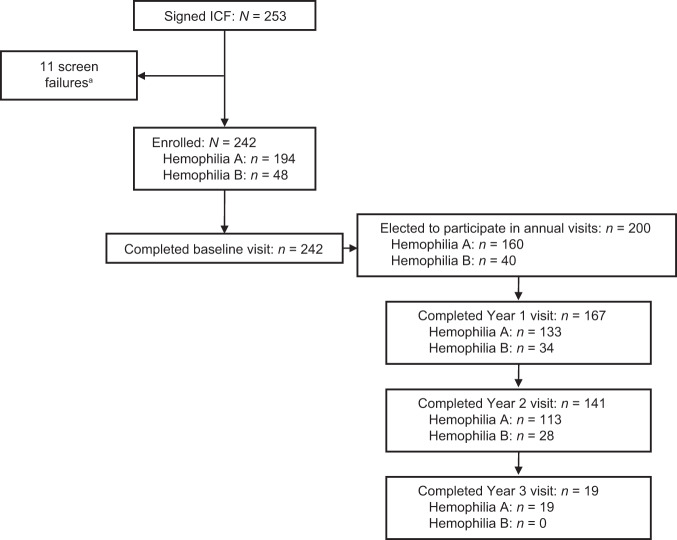
Participant disposition. ^a^Nine participants had personal laboratory evidence of having developed inhibitors to FVIII or FIX protein at any time (≥0.6 Bethesda units on any single test), one participant had a bleeding disorder other than hemophilia A or B, and one participant did not have established severe hemophilia A (plasma FVIII activity <1%) or B (plasma FIX activity ≤2%). ICF informed consent form.

Of 253 participants who were screened, 242 were enrolled in the study and had blood drawn at baseline (hemophilia A, *n* = 194; hemophilia B, *n* = 48). Baseline characteristics for these participants are shown in Table 1. In total, 200 participants elected to participate in annual visits (Fig. 1). The percentages of participants enrolling in the study, electing to complete annual visits, and completing the Year 1 and 2 visits were similar for hemophilia A and B. All participants who completed the Year 3 visit (*n* = 19) had hemophilia A. The study was terminated by the sponsor before most participants reached the Year 3 visit. As a result, 194 of 242 (80.2%) participants discontinued the study, of whom 124 (63.9%) discontinued because of study termination and 70 participants discontinued before the study was terminated. Of these, 28/194 (14.4%) discontinued because of withdrawal by the participant, 13/194 (6.7%) discontinued because of physician decision, and 29/194 (14.9%) discontinued for other reasons. Participant disposition by country is shown in Supplementary Fig. S2.

**Table 1:** Baseline characteristics.

	Hemophilia A (*n* = 194)	Hemophilia B (*n* = 48)	Total (*N* = 242)
Mean (SD) age at informed consent (years)	34.5 (11.3)	38.4 (11.7)	35.3 (11.4)
Ethnicity, *n* (%)			
Hispanic or Latino	10 (5.2)	1 (2.1)	11 (4.5)
Not Hispanic or Latino	116 (59.8)	25 (52.1)	141 (58.3)
Not disclosed	68 (35.1)	22 (45.8)	90 (37.2)
Race, *n* (%)			
White	114 (58.8)	23 (47.9)	137 (56.6)
Black/African American	5 (2.6)	2 (4.2)	7 (2.9)
Asian	4 (2.1)	0 (0.0)	4 (1.7)
Other	2 (1.0)	1 (2.1)	3 (1.2)
Not disclosed	69 (35.6)	22 (45.8)	91 (37.6)
Disease severity, *n* (%)			
Severe	194 (100)	30 (62.5)	224 (92.6)
Moderate	0 (0.0)	18 (37.5)	18 (7.4)
Historical factor VIII (IU/dl), *n* (%)			
<1	194 (100)	–	–
≥1	0 (0.0)	–	–
Historical factor IX (IU/dl), *n* (%)			
<1	–	30 (62.5)	–
1–2	–	18 (37.5)	–

*SD* standard deviation.

### Prevalence of NAbs

**Fig. 2:**
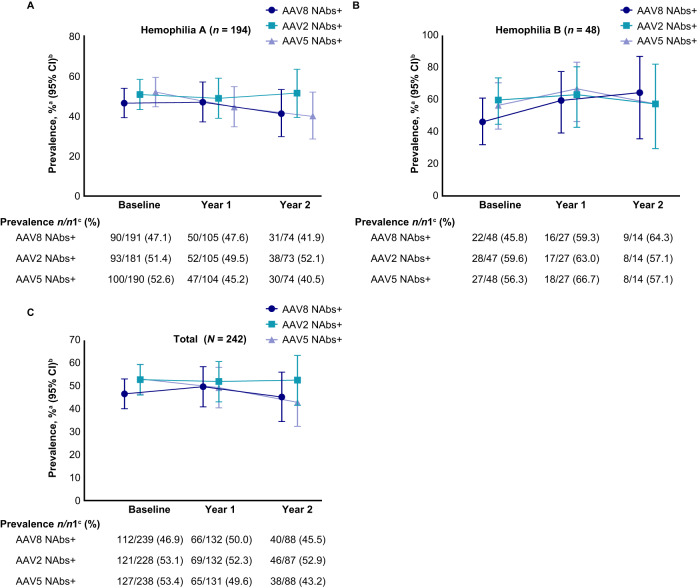
Prevalence of NAbs to AAV8, AAV2, and AAV5. Prevalence of NAbs to AAV8, AAV2, and AAV5 in participants with **A** hemophilia A, **B** hemophilia B, and **C** total number of participants. Serology samples were all collected on a similar schedule, the staggered display is for graphical clarity only. ^a^Prevalence is defined as the percentage of participants who tested positive for NAb (equal to or greater than the NAb assay cut point) or negative (less than the NAb assay cut point) at the minimum required dilution (1:5) to the specific AAV serotype in a group. ^b^Binomial exact Clopper-Pearson method was used to calculate 95% CIs. ^c^*n* represents the total number of participants who had positive NAb titers at a visit in a group. *n*1 represents the total number of participants with NAb titer results at that visit.

The seroprevalence of NAbs to AAV at baseline, Year 1, and Year 2 of the study is summarized in Fig. 2. Owing to the low number of participants remaining in the study at Year 3, these data have not been included in Fig. 2, but have been provided in Supplementary Table S1. Approximately half of the participants had NAbs to any AAV serotype at baseline (AAV8, 46.9%; AAV2, 53.1%; AAV5, 53.4%). The percentages of participants with NAbs to AAV at Year 1 and Year 2 were consistent with the percentages at baseline.

**Fig. 3:**
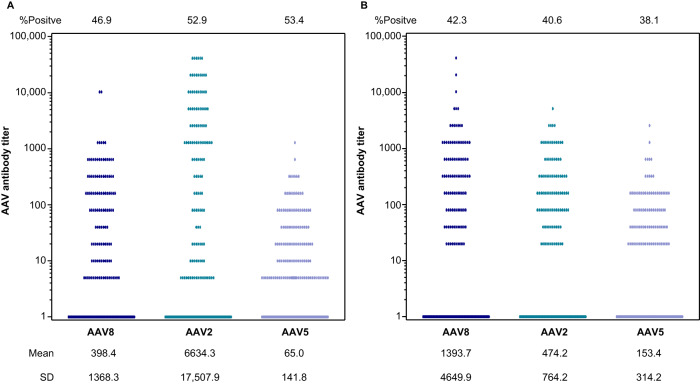
NAb and BAb titers at baseline. **A** NAb titers and **B** BAb titers in enrolled participants at baseline. An NAb value of 163,840 for one participant (AAV2) has been removed because it was too high and skewed the output.

NAb titers for AAV8, AAV2, and AAV5 at baseline are shown in Fig. 3. The percentages of participants in whom AAV8, AAV2, or AAV5 NAbs were not detected (titers <1:5) are summarized in Fig. 2. At baseline, nearly half of the participants (46.9%) tested positive for AAV8 NAbs. A slightly higher percentage of participants tested positive for AAV2 (53.1%) or AAV5 (53.4%) NAbs at baseline. The percentages of participants with detectable NAbs at Year 1 and Year 2 were similar to those at baseline.

One participant who did not have NAbs to AAV8 and AAV5 at baseline and had a low titer (20) of AAV2 NAbs received AAV5 gene therapy in another sponsored clinical trial and subsequently developed NAb titers ≥5120 for all three AAV serotypes (AAV8 titer, 5120; AAV2 titer, 40,960; AAV5 titer, 655,360) at Year 1.

Country-specific prevalence of NAbs at baseline is presented in Supplementary Fig. S3. The prevalence of AAV8 NAbs ranged from 33.3% in Italy to 52.7% in France, the prevalence of AAV2 NAbs ranged from 35.3% in Italy to 59.8% in France, and the prevalence of AAV5 NAbs ranged from 33.3% in Italy to 65.0% in Spain.

The co-prevalence of NAbs to each AAV serotype at each visit is summarized in Table 2. At baseline, ~40% of all participants had co-prevalent NAbs to two AAV serotypes and 38.2% had co-prevalent NAbs to all three AAV serotypes. More than 70% of participants who had NAbs to any one AAV serotype had NAbs to one or both of the other AAV serotypes. Participants who tested positive for AAV8 NAbs had higher rates of co-prevalence of NAbs to AAV2, AAV5, or both than participants who tested positive for AAV5 or AAV2 NAbs. Co-prevalence results at Year 1 and Year 2 were similar to baseline.

**Table 2:** Cross-tabulation of NAb status to AAV2, AAV8, and AAV5 at baseline in decreasing order of frequency.

	AAV8 NAb^a^	AAV2 NAb^a^	AAV5 NAb^a^	*n* (%)^b^	Cumulative, *n* (%)^b^
Overall	+	+	+	87 (38.2)	87 (38.2)
	–	–	–	71 (31.1)	158 (69.3)
	–	+	–	26 (11.4)	184 (80.7)
	–	–	+	23 (10.1)	207 (90.8)
	+	–	+	7 (3.1)	214 (93.9)
	+	–	–	6 (2.6)	220 (96.5)
	+	+	–	5 (2.2)	225 (98.7)
	–	+	+	3 (1.3)	228 (100)
Hemophilia A	+	+	+	65 (35.9)	65 (35.9)
	–	–	–	56 (30.9)	121 (66.9)
	–	+	–	21 (11.6)	142 (78.5)
	–	–	+	19 (10.5)	161 (89.0)
	+	–	+	7 (3.9)	168 (92.8)
	+	–	–	6 (3.3)	174 (96.1)
	+	+	–	5 (2.8)	179 (98.9)
	–	+	+	2 (1.1)	181 (100)
Hemophilia B	+	+	+	22 (46.8)	22 (46.8)
	–	–	–	15 (31.9)	37 (78.7)
	–	+	–	5 (10.6)	42 (89.4)
	–	–	+	4 (8.5)	46 (97.9)
	–	+	+	1 (2.1)	47 (100)

+ indicates positive and – indicates negative.

*AAV* adeno-associated virus, *NAb* neutralizing antibody.

^a^Positive (negative) NAb titers are defined as the titers equal to or greater than (less than) the NAb assay cut point (5) at the minimum required dilution (1:5).

^b^The percentages are based on the number of enrolled participants with all three NAb titer nonmissing results.

### Prevalence of BAbs

**Fig. 4:**
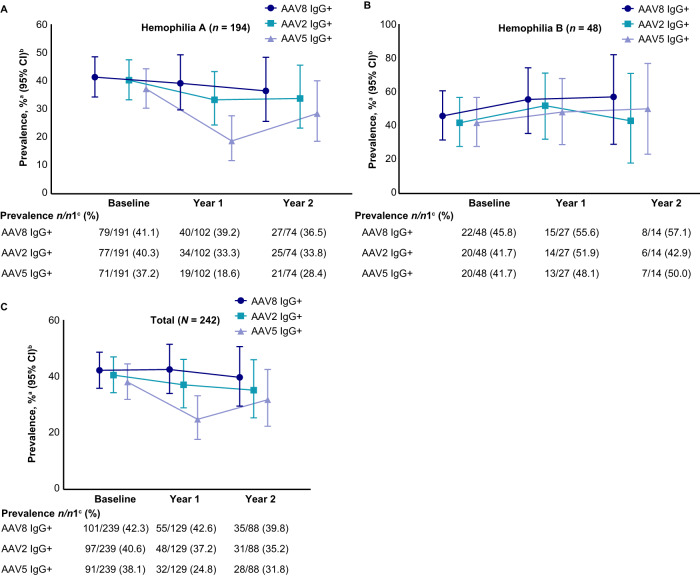
Prevalence of IgG BAbs to AAV8, AAV2, and AAV5. Prevalence of BAbs to AAV8, AAV2, and AAV5 in participants with **A** hemophilia A, **B** hemophilia B, and **C** total number of participants. ^a^Prevalence is defined as the percentage of participants who tested positive (equal to or greater than screening assay floating cut point) or negative (less than the screening assay floating cut point) at the minimum required dilution (1:20) in a specific AAV serotype group. ^b^Binomial exact Clopper-Pearson method was used to calculate 95% CIs. ^c^*n* represents the total number of participants who had positive BAb titers at a visit in a group. *n*1 represents the total number of participants with BAb titer results at that visit. + indicates positive.

The prevalence of IgG BAbs to AAV8, AAV2, and AAV5 is summarized in Fig. 4. Approximately 40% of participants tested positive for IgG Abs to any of the AAV serotypes at baseline (AAV8, 42.3%; AAV2, 40.6%; AAV5, 38.1%). The prevalence of IgG BAbs to AAV5 was lower in subsequent years compared with baseline (Year 1, 24.8%; Year 2, 31.8%). Country-specific prevalence of BAbs at baseline are presented in Supplementary Fig. S4, with associations between BAbs and NAbs shown in Supplementary Table S2. Few participants (7.5%) had IgM BAbs to AAV8 at baseline. The prevalence of IgM BAbs to AAV8 was 15.5% at Year 1 and 8.0% at Year 2.

At baseline, ~33% of participants had co-prevalent BAbs to two AAV serotypes, and 31.4% had co-prevalent BAbs to all three AAV serotypes. Approximately 80% of participants who had BAbs to any one AAV serotype had BAbs to one other AAV serotype, and ~75% had BAbs to the other two AAV serotypes. Participants who tested positive for AAV5 BAbs had higher co-prevalence rates of BAbs to AAV8, AAV2, or both than participants who tested positive for BAbs to AAV2 or AAV8. The co-prevalence of BAbs to AAV8 and AAV5, AAV2 and AAV5, and all three AAV serotypes decreased to 22.5% at Year 1. Participants who were AAV5 positive for BAbs at Year 1 had a 90.6% co-prevalence of BAbs to the other AAV serotypes. At Year 2, ~30% of participants had co-prevalence of BAbs to two or all three AAV serotypes; BAb co-prevalence was highest for participants who were AAV5 BAb positive (>90%).

**Fig. 5:**
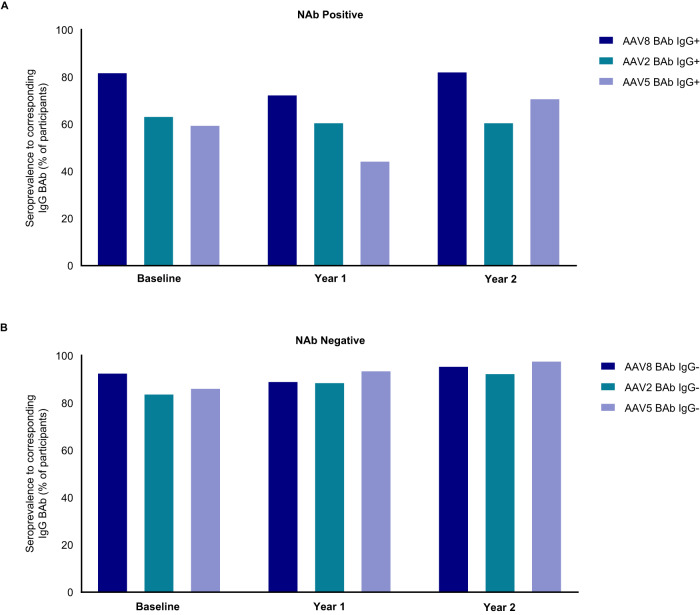
Co-prevalence of NAb and corresponding IgG BAb. Percentage of total study participants with co-prevalence of NAb and corresponding IgG BAb with **A** NAb positive and **B** NAb negative titers. Longitudinal follow-up is presented.

BAbs and NAbs are orthogonal determinations of immunity against capsid serotypes that will effectively inhibit vector transduction. As shown in Table 3 and Fig. 5, participants who are positive for NAbs were also positive for IgG BAbs to the same serotype and other serotypes at baseline (Supplementary Table S2). However, the baseline correlation between capsid specific NAb and BAb positivity was more frequently observed with the AAV8 serotype (82.1%) compared to AAV2 (63.6%) or AAV5 (59.8%). A negative NAb was more often associated with a negative BAb IgG at baseline for the same capsid for all serotypes tested (92.9%, 84.1%, and 86.5% for AAV8, AAV2, and AAV5, respectively). These correlations remained similar over the follow-up intervals and no discordant trends were observed between the hemophilia A and B populations.

**Table 3:** AAV NAb positive and NAb negative with corresponding IgG BAb capsid seropositivity.

	AAV8 NAb+		AAV2 NAb+		AAV5 NAb+	
	BAb IgG+	BAb IgG−	BAb IgG+	BAb IgG−	BAb IgG+	BAb IgG−
Baseline	92 (82.1)	20 (17.9)	77 (63.6)	44 (36.4)	76 (59.8)	51 (40.2)
Year 1	48 (72.7)	15 (22.7)	42 (60.9)	25 (36.2)	29 (44.6)	34 (52.3)
Year 2	33 (82.5)	7 (17.5)	28 (60.9)	18 (39.1)	27 (71.1)	11 (28.9)
	**AAV8 NAb−**		**AAV2 NAb−**		**AAV5 NAb−**	
	**BAb IgG**+	**BAb IgG−**	**BAb IgG**+	**BAb IgG−**	**BAb IgG**+	**BAb IgG−**
Baseline	9 (7.1)	118 (92.9)	17 (15.9)	90 (84.1)	15 (13.5)	96 (86.5)
Year 1	7 (10.6)	113 (89.4)	6 (9.5)	56 (88.9)	3 (4.5)	62 (93.9)
Year 2	2 (4.2)	46 (95.8)	3 (7.3)	38 (92.7)	1 (2.0)	49 (98.0)

+ indicates positive and – indicates negative.

*AAV* adeno-associated virus, *BAb* binding antibody, *Ig* immunoglobulin, *NAb* neutralizing antibody.

**Fig. 6:**
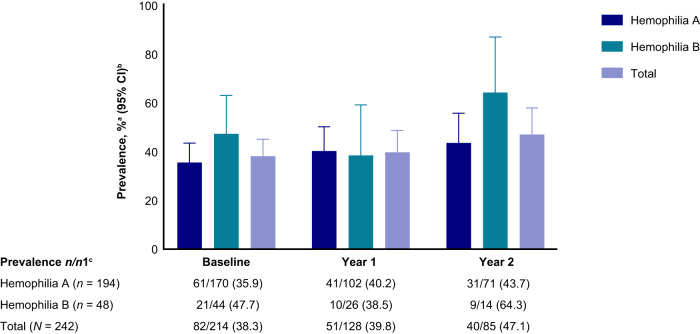
Prevalence of T cell–mediated immune response to AAV8 peptides. ^a^Prevalence is defined as the percentage of participants who tested positive (equal to or greater than screening assay floating cut point) or negative (less than the screening assay floating cut point). ^b^Binomial exact Clopper-Pearson method was used to calculate 95% CIs. ^c^*n* represents the total number of participants who presented with antigen-specific response (overall or by pool) at each visit. *n*1 represents the total number of participants with ELISpot assay titer results at that visit.

Cell-mediated immunity to AAV8 antigens is summarized in Fig. 6. At baseline, 38.3% of participants had a T-cell response to at least one peptide pool, and 6.5% had T-cell responses to more than one pool. T-cell responses were most common for Pool 3 (30.0%), and the most common multiple pool response was to Pools 2 and 3 (3.8%). Few responses (0.5%) were seen to all three pools. Antigen-specific response fluctuated over time; at Year 2, 47.1% of participants had a response to at least one pool, 41.2% had a response to Pool 3, and 11.8% had a response to multiple pools. No participants had a response to all three pools. A positive ELISpot assay did not correlate with the presence of NAbs from wild-type exposure.

### AAV seroconversion and titer fluctuations

Among participants with available NAb results at Year 1 for each of the three AAV serotypes, ~10% of participants who tested negative for NAbs at baseline were seropositive at Year 1 (Table 4). At Year 2, no participants who tested negative for AAV8 NAbs at baseline seroconverted, whereas 13.9% of AAV2 Ab-negative participants and 7.9% of AAV5 Ab-negative participants seroconverted. The percentage of participants who tested positive for NAbs at baseline who then subsequently tested negative at Year 1 or Year 2 was higher for AAV5 (Year 1, 21.6%; Year 2, 30.0%) than for AAV2 (Year 1, 13.8%; Year 2, 17.8%) or AAV8 (Year 1, 10.6%; Year 2, 16.7%).

**Table 4:** NAb seroconversion.

	NAb status at annual visits, *n* (%)^a^					
	Year 1			Year 2		
NAb status at baseline	Negative	Positive	Missing^b^	Negative	Positive	Missing^b^
Hemophilia A (*n* = 138)^c^						
AAV8 NAb+ (*n* = 66)	7 (14.0)	43 (86.0)	16	8 (20.5)	31 (79.5)	27
AAV8 NAb– (*n* = 71)	48 (87.3)	7 (12.7)	16	35 (100)	0	36
AAV2 NAb+ (*n* = 64)	5 (10.9)	41 (89.1)	18	7 (19.4)	29 (80.6)	28
AAV2 NAb– (*n* = 65)	47 (90.4)	5 (9.6)	13	27 (84.4)	5 (15.6)	33
AAV5 NAb+ (*n* = 73)	13 (24.1)	41 (75.9)	19	14 (34.1)	27 (65.9)	32
AAV5 NAb– (*n* = 63)	44 (89.8)	5 (10.2)	14	30 (90.9)	3 (9.1)	30
Hemophilia B (*n* = 35)^c^						
AAV8 NAb+ (*n* = 18)	0	16 (100)	2	0	9 (100)	9
AAV8 NAb– (*n* = 17)	11 (100)	0	6	5 (100)	0	12
AAV2 NAb+ (*n* = 22)	4 (21.1)	15 (78.9)	3	1 (11.1)	8 (88.9)	13
AAV2 NAb– (*n* = 12)	6 (85.7)	1 (14.3)	5	4 (100)	0	8
AAV5 NAb+ (*n* = 22)	3 (15.0)	17 (85.0)	2	1 (11.1)	8 (88.9)	13
AAV5 NAb– (*n* = 13)	6 (85.7)	1 (14.3)	6	5 (100)	0	8
Total (*N* = 173)^c^						
AAV8 NAb+ (*n* = 84)	7 (10.6)	59 (89.4)	18	8 (16.7)	40 (83.3)	36
AAV8 NAb– (*n* = 88)	59 (89.4)	7 (10.6)	22	40 (100)	0	48
AAV2 NAb+ (*n* = 86)	9 (13.8)	56 (86.2)	21	8 (17.8)	37 (82.2)	41
AAV2 NAb– (*n* = 77)	53 (89.8)	6 (10.2)	18	31 (86.1)	5 (13.9)	41
AAV5 NAb+ (*n* = 95)	16 (21.6)	58 (78.4)	21	15 (30.0)	35 (70.0)	45
AAV5 NAb– (*n* = 76)	50 (89.3)	6 (10.7)	20	35 (92.1)	3 (7.9)	38

+ indicates positive and – indicates negative.

*AAV* adeno-associated virus, *NAb* neutralizing antibody.

^a^*n* represents the total number of participants who had negative or positive NAb titers at a follow-up visit. The percentages are based on the total number of participants who had a negative or positive NAb titer at baseline and had results for annual visits.

^b^Missing data owing to data not being collected, data collected but not reported, or participants’ not being available for this specific visit.

^c^*n*/*N* is the number of participants with any or a specific NAb result available at baseline among participants who enrolled for annual visits.

NAb titers for any of the three capsid antigens that fluctuated over the trial follow-up period from positive values to negative values (<5) occurred in 37 participants. In almost all cases, variation was within one to three titration steps from the initial screening value (values of 1:5 to 1:20 could be read at subsequent visits as negative [<5]) and corresponded to similar relative titer fluctuations with the respective BAb Igs. Fluctuation of Ab titers within this range is considered an acceptable bioanalytical variation.

Among participants who had BAb results at Year 1, <10% of participants who tested negative for NAbs at baseline were seropositive at Year 1 for each of the three AAV serotypes (Table 5). The percentage of participants who tested positive for AAV IgG BAbs at baseline and negative at Year 1 was higher for AAV5 (32.6%) than for AAV2 (15.1%) or AAV8 (3.9%). At both Year 1 and Year 2, <10% participants who tested negative for NAbs for any two or all three AAV serotypes seroconverted to become Ab positive for those serotypes. Approximately 90% of participants with NAb data at Year 1 and ~80% with NAb data at Year 2 continued to have co-prevalence of NAbs for AAV serotypes seen at baseline.

**Table 5:** Binding antibody seroconversion.

	Binding antibody status at annual visits, *n* (%)^a^					
	Year 1			Year 2		
Binding antibody status at baseline	Negative	Positive	Missing	Negative	Positive	Missing
Hemophilia A (*n* = 138)^b^						
AAV8 IgG+ (*n* = 53)	2 (5.6)	34 (94.4)	17	6 (18.8)	26 (81.3)	21
AAV8 IgG– (*n* = 84)	60 (90.9)	6 (9.1)	18	41 (97.6)	1 (2.4)	42
AAV8 IgM+ (*n* = 8)	2 (28.6)	5 (71.4)	1	3 (75.0)	1 (25.0)	4
AAV8 IgM– (*n* = 129)	84 (88.4)	11 (11.6)	34	66 (94.3)	4 (5.7)	59
AAV2 IgG+ (*n* = 53)	7 (17.9)	32 (82.1)	14	10 (32.3)	21 (67.7)	22
AAV2 IgG– (*n* = 84)	61 (96.8)	2 (3.2)	21	39 (90.7)	4 (9.3)	41
AAV5 IgG+ (*n* = 47)	14 (43.8)	18 (56.3)	15	10 (34.5)	19 (65.5)	18
AAV5 IgG– (*n* = 90)	69 (98.6)	1 (1.4)	20	43 (95.6)	2 (4.4)	45
Hemophilia B (*n* = 35)^b^						
AAV8 IgG+ (*n* = 17)	0	15 (100)	2	0	8 (100)	9
AAV8 IgG– (*n* = 18)	12 (100)	0	6	6 (100)	0	12
AAV8 IgM+ (*n* = 2)	0	1 (100)	1	1 (100)	0	1
AAV8 IgM– (*n* = 33)	23 (88.5)	3 (11.5)	7	11 (84.6)	2 (15.4)	20
AAV2 IgG+ (*n* = 15)	1 (7.1)	13 (92.9)	1	0	6 (100)	9
AAV2 IgG– (*n* = 20)	12 (92.3)	1 (7.7)	7	8 (100)	0	12
AAV5 IgG+ (*n* = 16)	1 (7.1)	13 (92.9)	2	0	6 (100)	10
AAV5 IgG– (*n* = 19)	13 (100)	0	6	7 (87.5)	1 (12.5)	11
Total (*N* = 173)^b^						
AAV8 IgG+ (*n* = 70)	2 (3.9)	49 (96.1)	19	6 (15.0)	34 (85.0)	30
AAV8 IgG– (*n* = 102)	72 (92.3)	6 (7.7)	24	47 (97.9)	1 (2.1)	54
AAV8 IgM+ (*n* = 10)	2 (25.0)	6 (75.0)	2	4 (80.0)	1 (20.0)	5
AAV8 IgM– (*n* = 162)	107 (88.4)	14 (11.6)	41	77 (92.8)	6 (7.2)	79
AAV2 IgG+ (*n* = 68)	8 (15.1)	45 (84.9)	15	10 (27.0)	27 (73.0)	31
AAV2 IgG– (*n* = 104)	73 (96.1)	3 (3.9)	28	47 (92.2)	4 (7.8)	53
AAV5 IgG+ (*n* = 63)	15 (32.6)	31 (67.4)	17	10 (28.6)	25 (71.4)	28
AAV5 IgG– (*n* = 109)	82 (98.8)	1 (1.2)	26	50 (94.3)	3 (5.7)	56

+ indicates positive and – indicates negative.

*AAV* adeno-associated virus, *Ig* immunoglobulin.

^a^*n* represents the total number of participants who had negative or positive binding antibody titers at a follow-up visit. The percentages are based on the total number of participants who had a negative or positive binding antibody titer at baseline and had results for annual visits.

^b^*n/N* is the number of participants with any or a specific binding antibody result available at baseline among participants who enrolled for annual visits.

At both Year 1 and Year 2, <7% of participants who tested negative for BAbs for any two or all three AAV serotypes seroconverted to become Ab positive for those serotypes. At Year 1, 95.3% of participants continued to have co-prevalence of AAV8 and AAV2 BAbs, but the percentage with co-prevalence decreased to 71.8% for AAV8 and AAV5 Abs, 75.7% for AAV2 and AAV5 Abs, and 77.8% for AAV8, AAV2, and AAV5 Abs. Approximately 85% of participants with results at Year 2 continued to have the co-prevalence of BAbs for AAV serotypes seen at baseline.

Among participants who had no T-cell response to any pool at baseline, ~40% of those with follow-up results tested positive to one or more pools (30.9% tested positive to one pool and 7.4% to two pools at Year 1, and 28.3% to one pool and 13.2% to two pools at Year 2). Among participants with a response to one pool at baseline, 56.5% at Year 1 and 47.1% at Year 2 had no response to any pool.

## Discussion

Findings from this prospective global epidemiological study confirm the presence of significant preexisting humoral and cellular responses to AAV8, AAV2, and AAV5 in the adult male hemophilia population. Because gene therapy products are now approved for marketing authorization and commercial use in hemophilia, an understanding of long-term prevalence and persistence of immunity related to AAV vectors remains highly relevant.

Seroprevalence of NAbs (as defined by a titer ≥1:5 at baseline) was estimated at 46.9%, 53.1%, and 53.4% for AAV8, AAV2, and AAV5, respectively, in this study population. Therefore, ~50% of participants tested negative for NAbs and would be eligible for participation in a gene therapy study utilizing this cutoff value. The capsid-specific seroprevalence reported here is generally in line with ranges reported in other studies [12, 14, 15]. However, AAV5 NAb titers reported here are slightly higher than in some other reports [12], whereas the seroprevalence of NAbs against AAV2 (~40–50%) was lower, although a broad range of seroprevalences has been reported [12, 14, 16–18]. Because AAV seroprevalence varies by geographic region [12, 16, 18], comparisons across individual studies are challenging. In our study, AAV5 seroprevalence was higher in the United States and Spain compared with other regions, though we did not observe consistent differences between the European centers or by hemophilia type.

Comparing across studies is difficult for the reasons outlined earlier. When comparing the results of this report to others [12] we observe differences in positivity in the US, especially related to AAV5 seropositivity. The majority of our participants in the US were positive for AAV5 NAbs, compared to a lower proportion of the population reported by Klamroth et al. [12]. Assay variability remains one significant limitation when comparing results across studies using different methods, though our estimates are similar in some geographies (Italy for example). We also propose that different local metropolitan regions may have significantly different rates of seropositivity that is driven by multiple factors including racial and socioeconomic background [19], especially across a large geographic region like the US. Indeed, variability of other capsid seropositivity has been observed to vary by two-fold between different states in the US [20]. Therefore, it is important that local hemophilia treatment centers collect information on seroprevalence in their region for optimal understanding of gene therapy eligibility.

The relatively higher seroprevalence of AAV5 NAbs reported here should be placed in the context of other observations related to this capsid serotype [12], and with the understanding that no international positive control for AAV5 assays exists despite two approved gene therapies using this capsid [21–23]. In our study, we used a NAb assay as a transduction interference screen for AAV capsids. Some studies have demonstrated that anti-AAV5 NAbs interfere with gene transduction in nonhuman primates [24]; however, other studies have observed no relationship between the presence of pretreatment AAV5 NAbs and therapeutic efficacy, calling into question the significance of detecting preexisting immunity and the thresholds used for this method in vivo and in clinical trials [25, 26]. As gene therapies have already been approved for hemophilia A and B with an AAV5 vector for intravenous delivery, optimizing assays for this serotype is particularly relevant for clinical practice [21–23].

This study used a NAb titer cutoff of 1:5 for potential clinical trial eligibility. The role of such thresholds in determining participant selection (i.e., systematic exclusion of participants with titers ≥1:5) is subject to variability in assay methodology [27]. It is relevant to note that lower preexisting anti-capsid NAb titers (1:1) have been described as obstacles for optimal transgene expression in a hemophilia B clinical study [28]. Although our methodology is consistent with other reports, selection of assay components, pre-analytic variables, and specimen handling are known to impact the NAb assay [27, 29]. These variables are not routinely reported in clinical studies.

It is important to note that cutoff values drive seroprevalence estimations across different capsid serotypes. Our data demonstrates that higher mean values of anti-AAV antibodies were found in AAV2 NAbs compared to AAV8 and AAV5, and both AAV8 and AAV2 BAb mean titers were higher than AAV5 BAbs. AAV2 has been reported at higher prevalence in some studies, while AAV5 has been reported at lower prevalence vs AAV8 [12, 30]. A higher cutoff point from either assay would reduce the capsid-specific seropositivity of the study population, thus attention must be given to a robust determination of assay cutoff points, including careful reporting of methodology.

We also observed a higher NAb seroprevalence compared with the corresponding BAb seroprevalence. This can be partly explained by the different MRD and sensitivities of the cell-based assay used for detecting NAbs and the ligand-binding assay used for BAbs. NAb values may correlate with ligand-based assays, particularly IgG titers [30, 31], but BAbs also cross-react against other AAV serotypes [32]. Conversely, a lack of correlation between AAV5 BAbs and NAbs has also been reported [14]. Transduction interference functional assays are currently the preferred method to determine eligibility for AAV-based gene therapies. The significance of BAbs in the absence of NAb positivity and the potential impact on vector transduction remains unclear, but correlations between serotype specific NAbs and BAbs were observed in the majority of samples. As this study focused specifically on AAV8 as a planned gene therapy delivery vector, both IgG and IgM BAb values were assessed for this serotype. A small fraction of participants tested positive for IgM Abs against AAV8, and IgM titers remained stable throughout the multiyear follow-up. This is in line with other reports in healthy volunteers [30]. IgM titers are not used routinely in screening for gene therapy eligibility but are potentially reflective of recent wild-type exposure or preexisting innate immunity to vectors. Of note, preexisting IgM immunity has been reported to interfere with transduction of non-AAV viral vectors such as adenovirus [33].

We observed that immune responses to AAV serotypes were maintained throughout the follow-up interval, suggesting that seroprevalence remains largely stable over several years in this population. This is consistent with previous data on AAV serotypes showing stable Ab titers over a 6-month follow-up period [12].

Only 19 participants completed the Year 3 follow-up and results of seropositivity are shown in Supplementary Table S1. Although persistent positivity of NAbs is observed for all three capsids in this population, the small sample size prevents robust analysis. No participants with hemophilia B remained in the study at this timepoint. Additional long-term analysis of serotype positivity could inform the future use of AAV-based gene therapy in the adult population.

Long-term longitudinal data analyzing AAV seroprevalence are limited, although cross-sectional studies show that seropositivity tends to increase with age and other underlying immunologic status [12, 34, 35]. A study of seroprevalence to AAV over 6 years of follow-up in children and young adults with Duchenne muscular dystrophy, however, showed no change in anti-AAV Ab titers [36].

We also report that individual titers can fluctuate upon retesting in a small number of participants, which has significant implications for potential trial participation. Although assay variability is a factor in any screening test, eligibility for AAV therapies may be determined using only a single time point. Because NAb titers from wild-type exposure are often orders of magnitude lower than those observed after gene therapy treatment, the variability at the lower limit of quantification can have a direct impact on patients’ access to therapy. A single screening value may not be sufficient to determine the true serological status.

In this study, the prevalence of cell-mediated immunity in adults with hemophilia was determined using an IFN-γ–based ELISpot. We did not observe a correlation between a positive ELISpot assay and humoral immunity from wild-type AAV exposure, though approximately one-third of the study population had preexisting anti-AAV8 responses including those with NAbs against AAV8. This is in line with other studies and with the observation of ELISpot assay positivity against AAV8 and other AAV serotypes in healthy volunteers [30, 37–39]. Cell-mediated immunity remains difficult to assay in peripheral circulation. Some AAV gene therapy studies have described a correlation between ELISpot assay positivity, transaminase elevation, and loss of transgene expression after vector infusion, whereas others have found no association [11, 40]. Improvements in ELISpot assay methodology and handling are reported to improve assay performance and detection after therapeutic gene delivery [41]. The assay used in this study was based on IFN-γ, the primary cytokine associated with natural killer cells and T cells. Use of alternative cytokine-focused ELISpot assays (e.g., TNF-α) or flow cytometry cellular cytokine assays may provide additional information on the role of circulating T-cell populations that is relevant for AAV-based gene therapy [38, 40]. The majority of relevant capsid-specific T cells may be present in large amounts only in liver and spleen tissue. In a study of an AAV8 gene therapy in macaques, T-cell responses could be detected at the tissue level but not in peripheral circulation [42]. Therefore, the relevance of detecting a circulating lymphocyte using an ELISpot assay prior to gene delivery remains unclear.

We had one opportunistic finding in this study because one participant was treated with an AAV5-based gene therapy after enrollment in this study. This individual had high-titer NAbs against AAV5 in addition to cross-reactive titers to the other AAV serotypes. This finding highlights the real-world challenges with retreatment using AAV-based gene therapy, even if a different capsid serotype is used. Clinical trials are currently in progress that use therapeutic modalities methods to reduce preexisting NAb titers [43, 44]. If successful, these technologies could enable redosing of AAV gene therapy.

The present study has several limitations. First, because this study was also designed to enable participation in future interventional trials, there is potential for bias in the self-selection of participants who elected to participate in the annual follow-up. Some participants who tested positive for NAbs at baseline did not continue with the optional follow-up component of the study, while others who tested negative at screening joined interventional studies and were therefore ineligible for follow-up visits in this study. Similarly, this study was intended to be used by the trial sponsor to pre-screen participants for AAV8 based gene therapy. An AAV8-based hemophilia A gene therapy trial was in progress at the time that this study was conducted and may explain why few hemophilia B participants were approached and enrolled in some geographies like Spain.

Second, pediatric participants were not included in this study. Because gene therapy would have a major impact by preventing bleeds in early childhood, seroprevalence in patients under the age of 18 years is significant to the field. AAV NAb prevalence has consistently been reported as lower in pediatric participants [12, 45–47], but there are fewer longitudinal follow-up studies in a dedicated global pediatric cohort. Finally, local serologic prevalence and population subgroups were beyond the scope of the current study but are an avenue for more detailed future research. Over one-third of the study population did not report ethnicity data. Consequently, the impact of ethnicity on the seroprevalence of anti-AAV Abs could not be analyzed. A previous study in the United States has reported that ethnicity has an impact on AAV NAb seroprevalence [19]. This factor must be considered in the future to ensure equitable access to AAV-based gene therapies.

In conclusion, the results of this study confirm that adult male patients with severe hemophilia have significant preexisting humoral and cellular responses to AAV8, AAV2, and AAV5. NAbs remain a significant barrier to retreatment with existing viral vectors, although the impact of cellular immunity remains to be determined. Future research should focus on assay standardization and seroprevalence estimates in key patient subpopulations.

### Supplementary information

SUPPLEMENTARY INFORMATION

## Data Availability

The datasets, including the redacted study protocol, redacted statistical analysis plan, and individual participants’ data supporting the results of the study, will be made available after the publication of study results within 3 months from initial request to researchers who provide a methodologically sound proposal. The data will be provided after its de-identification, in compliance with applicable privacy laws, data protection, and requirements for consent and anonymization.
