# Supplementary material for: Multicenter assessment and longitudinal study of the prevalence of antibodies and related adaptive immune responses to AAV in adult males with hemophilia
Source: Gene Ther. 2024 Feb 14;31(5-6):273–84. doi: 10.1038/s41434-024-00441-5 (PMC11090810; doi:10.1038/s41434-024-00441-5)
Supplement: Supplementary file 1 — SUPPLEMENTARY INFORMATION [file 41434_2024_441_MOESM1_ESM.pdf]

TITLE: Multicenter assessment and longitudinal study of the prevalence of antibodies and related adaptive immune responses to AAV in adult males with hemophilia

RUNNING TITLE: Adaptive immune responses to AAV in hemophilia

AUTHORS: Ingrid Pabinger[1], Mila Ayash-Rashkovsky[2], Miguel Escobar[3], Barbara A Konkle[4,5], María Eva Mingot-Castellano[6], Eric S. Mullins[7], Claude Negrier[8], Luying Pan[2], Kavitha Rajavel[2], Brian Yan[2] and John Chapin[2]

AFFILIATIONS: [1]Clinical Division of Hematology and Hemostaseology, Department of Medicine I, Medical University of Vienna, Vienna, Austria. [2]Takeda Development Center Americas Inc, Cambridge, MA, USA. [3]University of Texas Health Science Center, McGovern Medical School and Gulf States Hemophilia and Thrombophilia Center, Houston, TX, USA. [4]BloodWorks Northwest, Seattle, WA, USA. [5]Division of Hematology, University of Washington School of Medicine, Seattle, WA, USA. [6]Hospital Regional Universitario de Málaga, Málaga, Spain, and Hospital Universitario Virgen del Rocio, Sevilla, Spain. [7]Division of Hematology, Cincinnati Children's Hospital Medical Center and University of Cincinnati-College of Medicine, Cincinnati, OH, USA. [8]UR4609 Hemostase & Thrombose, University Lyon 1, Lyon, France.

Correspondence: Kavitha Rajavel (kavitha.rajavel@takeda.com) and John Chapin (jchapin29@gmail.com)

Target journal: Gene Therapy

Abstract word count: 200/200

Text word count: 4178/8000

Tables/Figures: 10 Tables and Figures and 5 Supplemental Tables and Figures

References: 44

SUPPLEMENTARY INFORMATION

Supplementary Fig. S1 Study design. [a]Participants could elect to complete the study after the baseline blood draw or return to the hemophilia treatment center for annual blood draws.

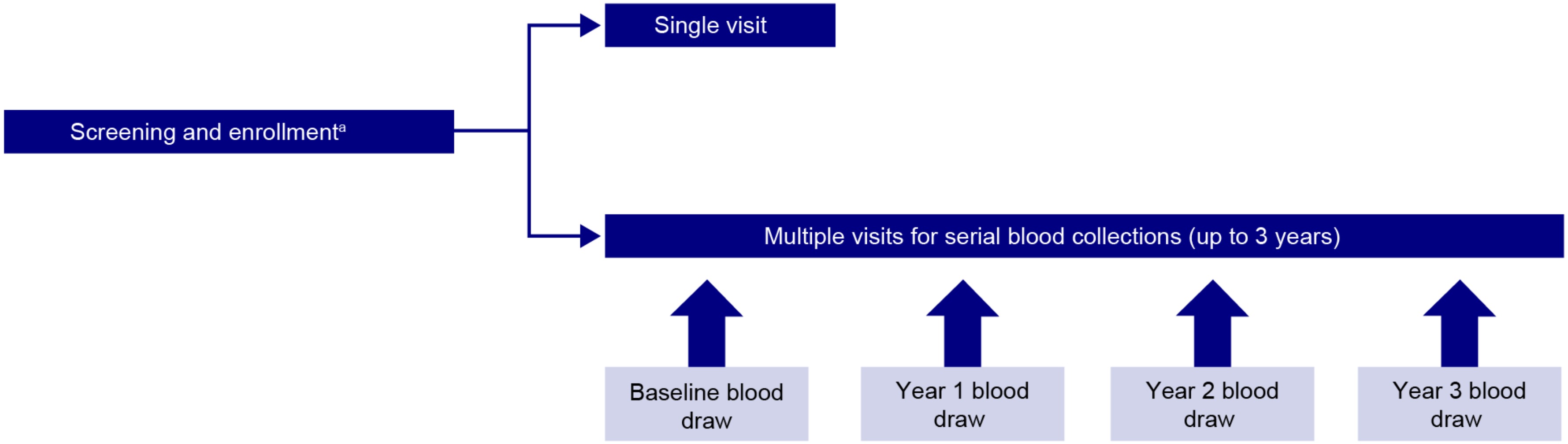

Supplementary Fig. S2 Patient disposition by country. *HA* hemophilia A, *HB* hemophilia B.

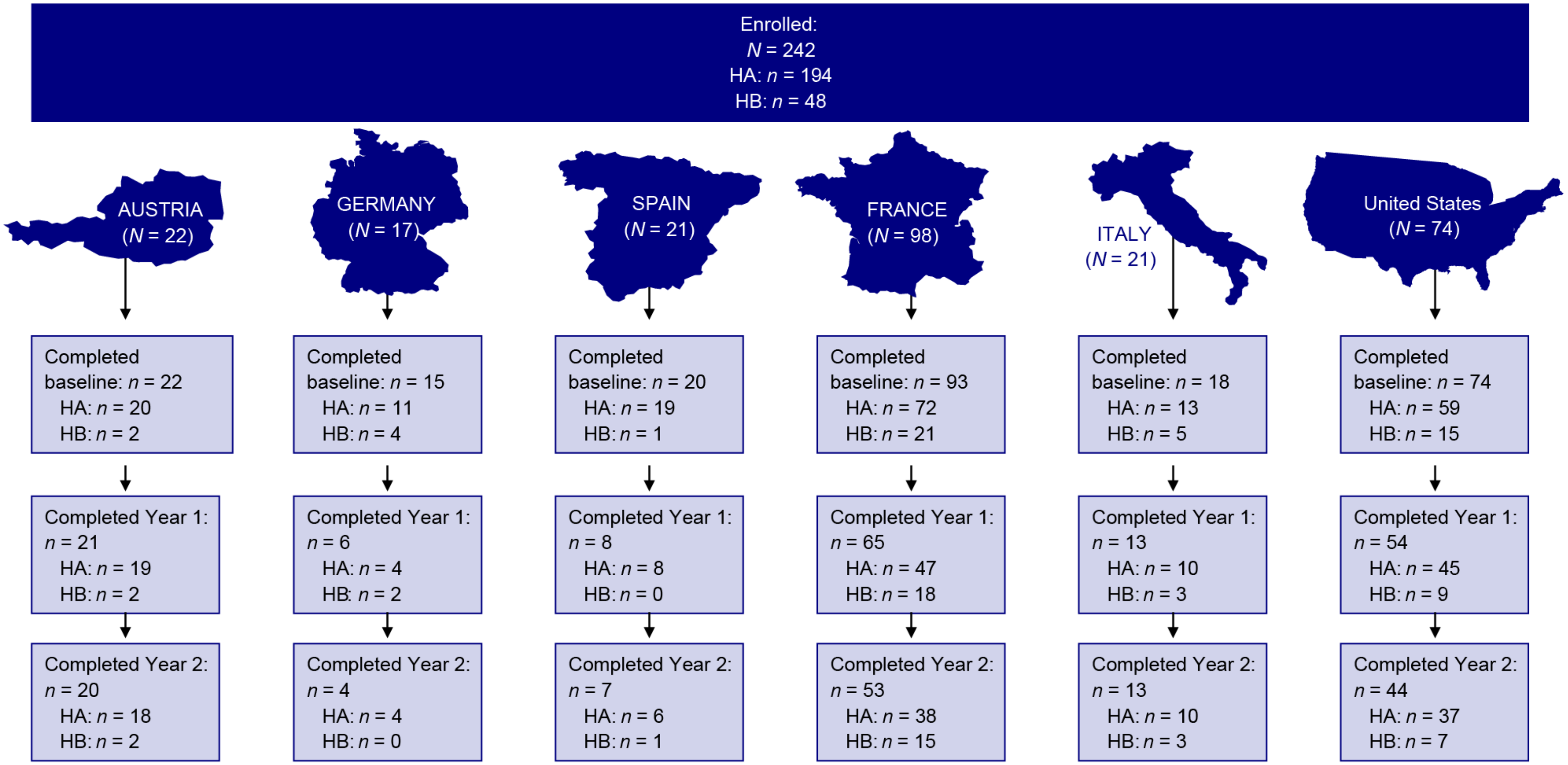

Supplementary Fig. S3 Prevalence of NAbs at baseline by country. Percentage of participants with **A** hemophilia A, **B** hemophilia B, and **C** total number of participants. *AAV* adeno-associated virus, *CI* confidence interval, *NAb* neutralizing antibody. *1 participant with hemophilia B was enrolled in Spain

A

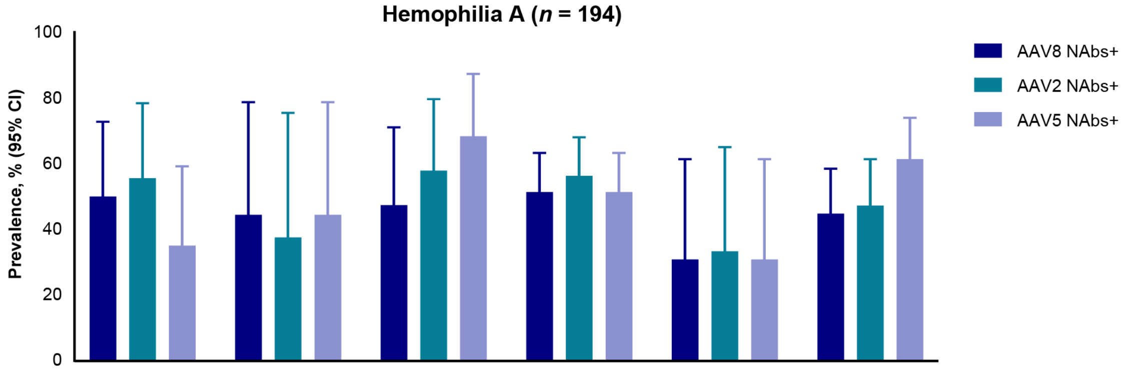

Prevalence, %	Austria	Germany	Spain	France	Italy	USA
AAV8 NAbs+	50.0	44.4	47.4	51.4	30.8	44.8
AAV2 NAbs+	55.6	37.5	57.9	56.3	33.3	47.2
AAV5 NAbs+	35.0	44.4	68.4	51.4	30.8	61.4

B

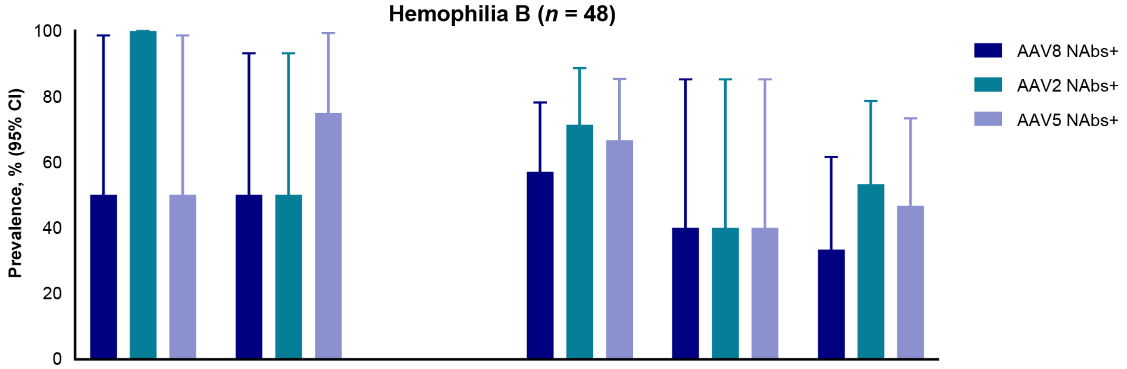

Prevalence, %	Austria	Germany	Spain*	France	Italy	USA
AAV8 NAbs+	50.0	50.0	0.0	57.1	40.0	33.3
AAV2 NAbs+	100.0	50.0	0.0	71.4	40.0	53.3
AAV5 NAbs+	50.0	75.0	0.0	66.7	40.0	46.7

C

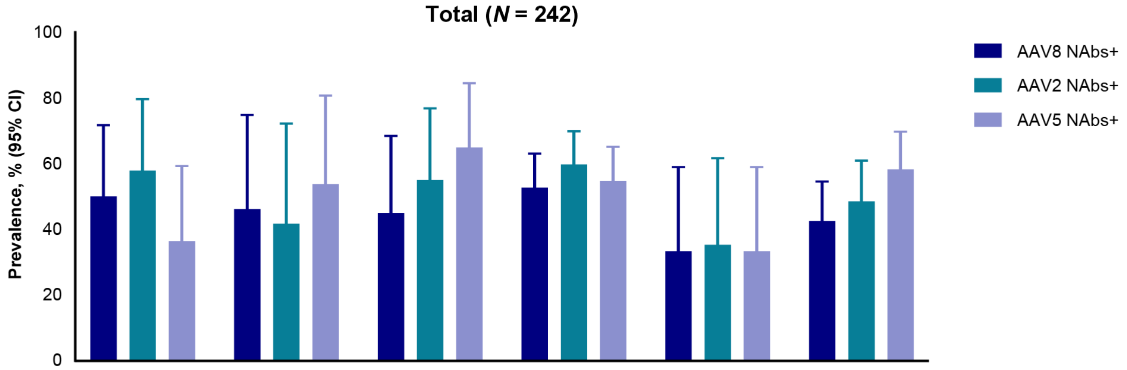

Prevalence, %	Austria	Germany	Spain*	France	Italy	USA
AAV8 NAbs+	50.0	46.2	45.0	52.7	33.3	42.5
AAV2 NAbs+	57.9	41.7	55.0	59.8	35.3	46.5
AAV5 NAbs+	36.4	53.8	65.0	54.8	33.3	58.3

Supplementary Fig. S4 Prevalence of binding antibodies at baseline by country. Percentage of participants with **A** hemophilia A, **B** hemophilia B, and **C** total number of participants. *AAV* adeno-associated virus, *CI* confidence interval, *IgG+* immunoglobulin G positive. *1 participant with hemophilia B was enrolled in Spain

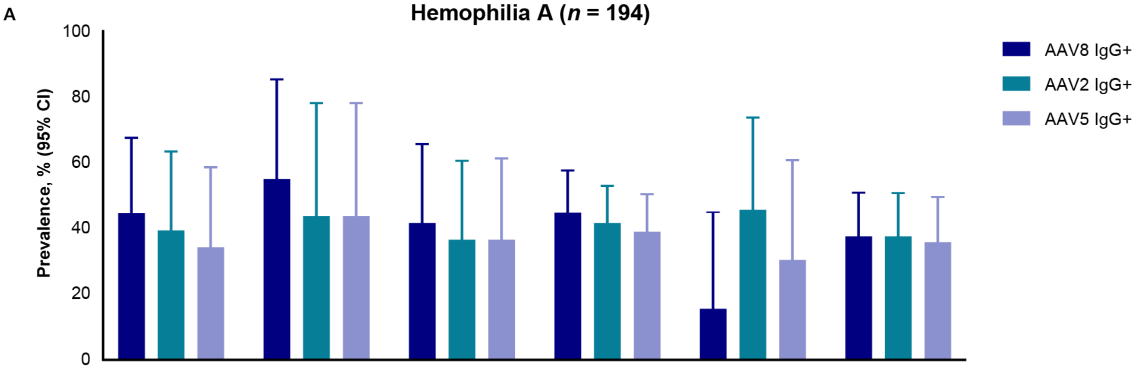

Prevalence, %	Austria	Germany	Spain	France	Italy	USA
AAV8 IgG+	45.0	55.6	42.1	45.8	15.4	37.9
AAV2 IgG+	40.0	44.4	36.8	41.7	46.2	37.9
AAV5 IgG+	35.0	44.4	36.8	38.9	30.8	36.2

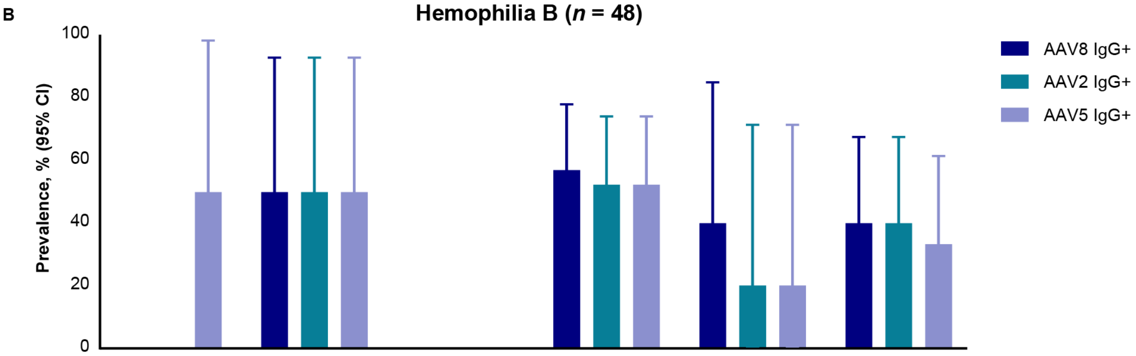

Prevalence, %	Austria	Germany	Spain*	France	Italy	USA
AAV8 IgG+	0.0	50.0	0.0	57.1	40.0	40.0
AAV2 IgG+	0.0	50.0	0.0	52.4	20.0	40.0
AAV5 IgG+	50.0	50.0	0.0	52.4	20.0	33.3

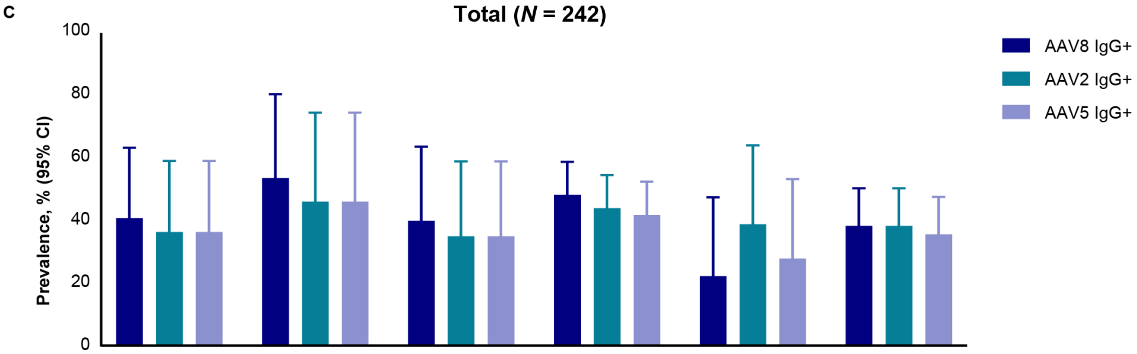

Prevalence, %	Austria	Germany	Spain*	France	Italy	USA
AAV8 IgG+	40.9	53.8	40.0	48.4	22.2	38.4
AAV2 IgG+	36.4	46.2	35.0	44.1	38.9	38.4
AAV5 IgG+	36.4	46.2	35.0	41.9	27.8	35.6

Supplementary Table 1. Prevalence of NAbs to AAV8, AAV2, and AAV5.

**Prevalence, *n*/*n*1[a], %[b] (95% CI)[c]**	**Hemophilia A (*n* = 194)**	**Hemophilia B (*n* = 48)**	**Total (*N* = 242)**
Baseline	194	48	242
AAV8 NAbs+	90/191, 47.1 (39.9, 54.5)	22/48, 45.8 (31.4, 60.8)	112/239, 46.9 (40.4, 53.4)
AAV2 NAbs+	93/181, 51.4 (43.9, 58.9)	28/47, 59.6 (44.3, 73.6)	121/228, 53.1 (46.4, 59.7)
AAV5 NAbs+	100/190, 52.6 (45.3, 59.9)	27/48, 56.3 (41.2, 70.5)	127/238, 53.4 (46.8, 59.8)
Year 1	133	34	167
AAV8 NAbs+	50/105, 47.6 (37.8, 57.6)	16/27, 59.3 (38.8, 77.6)	66/132, 50.0 (41.2, 58.8)
AAV2 NAbs+	52/105, 49.5 (39.6, 59.5)	17/27, 63.0 (42.4, 80.6)	69/132, 52.3 (43.4, 61.0)
AAV5 NAbs+	47/104, 45.2 (35.4, 55.3)	18/27, 66.7 (46.0, 83.5)	65/131, 49.6 (40.8, 58.5)
Year 2	113	28	141
AAV8 NAbs+	31/74, 41.9 (30.5, 53.9)	9/14, 64.3 (35.1, 87.2)	40/88, 45.5 (34.8, 56.4)
AAV2 NAbs+	38/73, 52.1 (40.0, 63.9)	8/14, 57.1 (28.9, 82.3)	46/87, 52.9 (41.9, 63.7)
AAV5 NAbs+	30/74, 40.5 (29.3, 52.6)	8/14, 57.1 (28.9, 82.3)	38/88, 43.2 (32.7, 54.2)
Year 3	19	0	19
AAV8 NAbs+	3/8, 37.5 (8.5, 75.5)	0/0, 0.0 (NE, NE)	3/8, 37.5 (8.5, 75.5)
AAV2 NAbs+	4/8, 50.0 (15.7, 84.3)	0/0, 0.0 (NE, NE)	4/8, 50.0 (15.7, 84.3)
AAV5 NAbs+	3/8, 37.5 (8.5, 75.5)	0/0, 0.0 (NE, NE)	3/8, 37.5 (8.5, 75.5)

+ indicates positive and – indicates negative.
[a]*n* represents the total number of participants who had positive NAb titers at a visit in a group. *n*1 represents the total number of participants with NAb titer results at that visit.
[b]Prevalence is defined as the percentage of participants who tested positive for NAbs (equal to or greater than the NAb assay cut point) or negative (less than the NAb assay cut point) at the minimum required dilution (1:5) to the specific AAV serotype in a group.
[c]Binomial exact Clopper-Pearson method was used to calculate 95% CIs.
AAV adeno-associated virus, *CI* confidence interval, *NAb* neutralizing antibody, *NE* not evaluable.

Supplementary Table 2. Association of NAbs and binding antibodies by hemophilia type and visit.

**Binding antibodies**	**AAV8 NAb, *n* (%)[b,c]**		**AAV2 NAb, *n* (%)[b,c]**		**AAV5 NAb, *n* (%)[b,c]**	
	**Negative[a] (*n* = 137)**	**Positive[d] (*n* = 119)**	**Negative[a] (*n* = 123)**	**Positive[d] (*n* = 139)**	**Negative[a] (*n* = 131)**	**Positive[d] (*n* = 136)**
**Hemophilia A**						
Baseline	101	90	88	93	90	100
AAV8 IgG						
Positive	8 (7.9)	71 (78.9)	14 (15.9)	62 (66.7)	13 (14.4)	66 (66.0)
Negative	93 (92.1)	19 (21.1)	74 (84.1)	31 (33.3)	77 (85.6)	34 (34.0)
AAV8 IgM						
Positive	6 (5.9)	8 (8.9)	3 (3.4)	9 (9.7)	4 (4.4)	10 (10.0)
Negative	95 (94.1)	82 (91.1)	85 (96.6)	84 (90.3)	86 (95.6)	90 (90.0)
AAV2 IgG						
Positive	17 (16.8)	60 (66.7)	15 (17.0)	59 (63.4)	19 (21.1)	58 (58.0)
Negative	84 (83.2)	30 (33.3)	73 (83.0)	34 (36.6)	71 (78.9)	42 (42.0)
AAV5 IgG						
Positive	13 (12.9)	58 (64.4)	14 (15.9)	55 (59.1)	14 (15.6)	57 (57.0)
Negative	88 (87.1)	32 (35.6)	74 (84.1)	38 (40.9)	76 (84.4)	43 (43.0)
Year 1	55	50	53	52	57	47
AAV8 IgG						
Positive	7 (12.7)	33 (66.0)	8 (15.1)	32 (61.5)	8 (14.0)	32 (68.1)
Negative	48 (87.3)	14 (28.0)	44 (83.0)	18 (34.6)	48 (84.2)	13 (27.7)
AAV8 IgM						
Positive	6 (10.9)	10 (20.0)	7 (13.2)	9 (17.3)	3 (5.3)	12 (25.5)
Negative	49 (89.1)	37 (74.0)	45 (84.9)	41 (78.8)	53 (93.0)	33 (70.2)
AAV2 IgG						
Positive	6 (10.9)	28 (56.0)	5 (9.4)	29 (55.8)	5 (8.8)	29 (61.7)
Negative	49 (89.1)	19 (38.0)	47 (88.7)	21 (40.4)	51 (89.5)	16 (34.0)
AAV5 IgG						
Positive	1 (1.8)	18 (36.0)	1 (1.9)	18 (34.6)	2 (3.5)	17 (36.2)
Negative	54 (98.2)	29 (58.0)	51 (96.2)	32 (61.5)	54 (94.7)	28 (59.6)
Year 2	43	31	35	38	44	30
AAV8 IgG						
Positive	2 (4.7)	25 (80.6)	1 (2.9)	26 (68.4)	4 (9.1)	23 (76.7)
Negative	41 (95.3)	6 (19.4)	34 (97.1)	12 (31.6)	40 (90.9)	7 (23.3)

AAV8 IgM						
Positive	2 (4.7)	3 (9.7)	2 (5.7)	3 (7.9)	2 (4.5)	3 (10.0)
Negative	41 (95.3)	28 (90.3)	33 (94.3)	35 (92.1)	42 (95.5)	27 (90.0)
AAV2 IgG						
Positive	4 (9.3)	21 (67.7)	3 (8.6)	22 (57.9)	5 (11.4)	20 (66.7)
Negative	39 (90.7)	10 (32.3)	32 (91.4)	16 (42.1)	39 (88.6)	10 (33.3)
AAV5 IgG						
Positive	0	21 (67.7)	0	21 (55.3)	1 (2.3)	20 (66.7)
Negative	43 (100)	10 (32.3)	35 (100)	17 (44.7)	43 (97.7)	10 (33.3)
**Hemophilia B**						
Baseline	26	22	19	28	21	27
AAV8 IgG						
Positive	1 (3.8)	21 (95.5)	1 (5.3)	21 (75.0)	1 (4.8)	21 (77.8)
Negative	25 (96.2)	1 (4.5)	18 (94.7)	7 (25.0)	20 (95.2)	6 (22.2)
AAV8 IgM						
Positive	2 (7.7)	2 (9.1)	1 (5.3)	3 (10.7)	2 (9.5)	2 (7.4)
Negative	24 (92.3)	20 (90.9)	18 (94.7)	25 (89.3)	19 (90.5)	25 (92.6)
AAV2 IgG						
Positive	2 (7.7)	18 (81.8)	2 (10.5)	18 (64.3)	2 (9.5)	18 (66.7)
Negative	24 (92.3)	4 (18.2)	17 (89.5)	10 (35.7)	19 (90.5)	9 (33.3)
AAV5 IgG						
Positive	1 (3.8)	19 (86.4)	0	20 (71.4)	1 (4.8)	19 (70.4)
Negative	25 (96.2)	3 (13.6)	19 (100)	8 (28.6)	20 (95.2)	8 (29.6)
Year 1	11	16	10	17	9	18
AAV8 IgG						
Positive	0	15 (93.8)	1 (10.0)	14 (82.4)	1 (11.1)	14 (77.8)
Negative	11 (100)	1 (6.3)	9 (90.0)	3 (17.6)	8 (88.9)	4 (22.2)
AAV8 IgM						
Positive	0	4 (25.0)	0	4 (23.5)	0	4 (22.2)
Negative	11 (100)	12 (75.0)	10 (100)	13 (76.5)	9 (100)	14 (77.8)
AAV2 IgG						
Positive	0	14 (87.5)	1 (10.0)	13 (76.5)	1 (11.1)	13 (72.2)
Negative	11 (100)	2 (12.5)	9 (90.0)	4 (23.5)	8 (88.9)	5 (27.8)
AAV5 IgG						
Positive	1 (9.1)	12 (75.0)	2 (20.0)	11 (64.7)	1 (11.1)	12 (66.7)
Negative	10 (90.9)	4 (25.0)	8 (80.0)	6 (35.3)	8 (88.9)	6 (33.3)

Year 2	5	9	6	8	6	8
AAV8 IgG						
Positive	0	8 (88.9)	0	8 (100)	1 (16.7)	7 (87.5)
Negative	5 (100)	1 (11.1)	6 (100)	0	5 (83.3)	1 (12.5)
AAV8 IgM						
Positive	1 (20.0)	1 (11.1)	1 (16.7)	1 (12.5)	1 (16.7)	1 (12.5)
Negative	4 (80.0)	8 (88.9)	5 (83.3)	7 (87.5)	5 (83.3)	7 (87.5)
AAV2 IgG						
Positive	0	6 (66.7)	0	6 (75.0)	0	6 (75.0)
Negative	5 (100)	3 (33.3)	6 (100)	2 (25.0)	6 (100)	2 (25.0)
AAV5 IgG						
Positive	0	7 (77.8)	1 (16.7)	6 (75.0)	0	7 (87.5)
Negative	5 (100)	2 (22.2)	5 (83.3)	2 (25.0)	6 (100)	1 (12.5)
**Total**	127	112	107	121	111	127
Baseline						
AAV8 IgG						
Positive	9 (7.1)	92 (82.1)	15 (14.0)	83 (68.6)	14 (12.6)	87 (68.5)
Negative	118 (92.9)	20 (17.9)	92 (86.0)	38 (31.4)	97 (87.4)	40 (31.5)
AAV8 IgM						
Positive	8 (6.3)	10 (8.9)	4 (3.7)	12 (9.9)	6 (5.4)	12 (9.4)
Negative	119 (93.7)	102 (91.1)	103 (96.3)	109 (90.1)	105 (94.6)	115 (90.6)
AAV2 IgG						
Positive	19 (15.0)	78 (69.6)	17 (15.9)	77 (63.6)	21 (18.9)	76 (59.8)
Negative	108 (85.0)	34 (30.4)	90 (84.1)	44 (36.4)	90 (81.1)	51 (40.2)
AAV5 IgG						
Positive	14 (11.0)	77 (68.8)	14 (13.1)	75 (62.0)	15 (13.5)	76 (59.8)
Negative	113 (89.0)	35 (31.3)	93 (86.9)	46 (38.0)	96 (86.5)	51 (40.2)
Year 1	66	66	63	69	66	65
AAV8 IgG						
Positive	7 (10.6)	48 (72.7)	9 (14.3)	46 (66.7)	9 (13.6)	46 (70.8)
Negative	59 (89.4)	15 (22.7)	53 (84.1)	21 (30.4)	56 (84.8)	17 (26.2)
AAV8 IgM						
Positive	6 (9.1)	14 (21.2)	7 (11.1)	13 (18.8)	3 (4.5)	16 (24.6)
Negative	60 (90.9)	49 (74.2)	55 (87.3)	54 (78.3)	62 (93.9)	47 (72.3)
AAV2 IgG						
Positive	6 (9.1)	42 (63.6)	6 (9.5)	42 (60.9)	6 (9.1)	42 (64.6)

Negative	60 (90.9)	21 (31.8)	56 (88.9)	25 (36.2)	59 (89.4)	21 (32.3)
AAV5 IgG						
Positive	2 (3.0)	30 (45.5)	3 (4.8)	29 (42.0)	3 (4.5)	29 (44.6)
Negative	64 (97.0)	33 (50.0)	59 (93.7)	38 (55.1)	62 (93.9)	34 (52.3)
Year 2	48	40	41	46	50	38
AAV8 IgG						
Positive	2 (4.2)	33 (82.5)	1 (2.4)	34 (73.9)	5 (10.0)	30 (78.9)
Negative	46 (95.8)	7 (17.5)	40 (97.6)	12 (26.1)	45 (90.0)	8 (21.1)
AAV8 IgM						
Positive	3 (6.3)	4 (10.0)	3 (7.3)	4 (8.7)	3 (6.0)	4 (10.5)
Negative	45 (93.8)	36 (90.0)	38 (92.7)	42 (91.3)	47 (94.0)	34 (89.5)
AAV2 IgG						
Positive	4 (8.3)	27 (67.5)	3 (7.3)	28 (60.9)	5 (10.0)	26 (68.4)
Negative	44 (91.7)	13 (32.5)	38 (92.7)	18 (39.1)	45 (90.0)	12 (31.6)
AAV5 IgG						
Positive	0	28 (70.0)	1 (2.4)	27 (58.7)	1 (2.0)	27 (71.1)
Negative	48 (100)	12 (30.0)	40 (97.6)	19 (41.3)	49 (98.0)	11 (28.9)

Positive binding antibody titers were defined as testing positive (equal to or greater than the screening assay floating cut point [20]) at the minimum required dilution (1:20). Negative binding antibody titers were defined as testing negative (less than the screening assay floating cut point [20]) at the minimum required dilution (1:20).

[a]*n* is number of negative NAb titer responses, defined as testing negative (less than the NAb assay cut point [5]) at the minimum required dilution (1:5). The percentages are based on the total number of patients with NAb results at each visit.

[b]Percentage is based on the total number of patients with neutralizing antibody results at each visit.

[c]*n* is the number of participants with an individual hemophilia type in the enrolled set that had binding antibody results at that visit.

[d]*n* is number of positive NAb titer responses, defined as testing positive (equal to or greater than the NAb assay cut point [5]) at the minimum required dilution (1:5).

AAV adeno-associated virus, *Ig* immunoglobulin, *NAb* neutralizing antibody.
